# Aesthetics and predictive processing: grounds and prospects of a fruitful encounter

**DOI:** 10.1098/rstb.2022.0410

**Published:** 2024-01-29

**Authors:** Jacopo Frascaroli, Helmut Leder, Elvira Brattico, Sander Van de Cruys

**Affiliations:** ^1^ Department of Psychology, University of Turin, 10124 Turin, Italy; ^2^ Faculty of Psychology and Cognitive Science Research Hub, University of Vienna, 1010 Vienna, Austria; ^3^ Center for Music in the Brain, Department of Clinical Medicine, Aarhus University, and Royal Academy of Music Aarhus/Aalborg, 8000 Aarhus, Denmark; ^4^ Department of Education, Psychology, Communication, University of Bari Aldo Moro, 70121 Bari, Italy; ^5^ Antwerp Social Lab, University of Antwerp, 2000 Antwerp, Belgium

**Keywords:** aesthetics, predictive processing, cognitive science, inference, meaning-making, affect

## Abstract

In the last few years, a remarkable convergence of interests and results has emerged between scholars interested in the arts and aesthetics from a variety of perspectives and cognitive scientists studying the mind and brain within the predictive processing (PP) framework. This convergence has so far proven fruitful for both sides: while PP is increasingly adopted as a framework for understanding aesthetic phenomena, the arts and aesthetics, examined under the lens of PP, are starting to be seen as important windows into our mental functioning. The result is a vast and fast-growing research programme that promises to deliver important insights into our aesthetic encounters as well as a wide range of psychological phenomena of general interest. Here, we present this developing research programme, describing its grounds and highlighting its prospects. We start by clarifying how the study of the arts and aesthetics encounters the PP picture of mental functioning (§1). We then go on to outline the prospects of this encounter for the fields involved: philosophy and history of art (§2), psychology of aesthetics and neuroaesthetics (§3) and psychology and neuroscience more generally (§4). The upshot is an ambitious but well-defined framework within which aesthetics and cognitive science can partner up to illuminate crucial aspects of the human mind.

This article is part of the theme issue ‘Art, aesthetics and predictive processing: theoretical and empirical perspectives’.

## 1. Linking aesthetics and the predictive mind

One of the most remarkable facts about the functioning of minds like ours is that, starting from the sparse and ambiguous stimulations hitting our sensory organs, we are able to reconstruct the meaningful world full of objects, people and ordered events that we inhabit. What is even more extraordinary is that in most cases we carry out this constructive activity so quickly and effortlessly that we are not aware of carrying it out, landing almost immediately on the most reasonable solutions for the puzzles posed to our senses. From this fleeting, messy, ever-changing bundle of light waves, sound waves and odour molecules, we immediately distil the figure of ‘our dog coming in through the front door', and from the mud left by her paws on our floor and the holes that have just appeared on our lawn we readily arrive at a plausible story that accounts for both facts. A way of capturing this wonderous ability of ours is to say that we are able to find structure in the world at different levels of abstraction and temporal scales (from discerning the objects that are producing a particular array of stimulations to detecting an underlying causal pattern in a series of events). The possibility of finding structure in this way is arguably the underpinning of much of perception, cognition, learning and other crucial mental capacities. The key idea underlying this theme issue is that understanding such possibility is the common task of two fields of inquiry that might seem very different at first but should really be seen as participating in the same endeavour: cognitive science and aesthetics. There is, in other words, a close relationship between the interdisciplinary study of the mind and the study of our experiences of beauty and art. Seeing this requires that we access a specific understanding of both our functioning as cognitive agents and the nature of our aesthetic encounters.

Let us start with this latter task. It would be difficult to group together the ample range of interests and preoccupations that have been ascribed to aesthetics, but there is certainly a crucial concern that has haunted it throughout its history, both before and after its formalization as a distinct philosophical endeavour in the eighteenth century, and up to the current trends in empirical aesthetics and neuroaesthetics. This is the question of why we find certain things aesthetically appealing, or—to use a word long fallen into philosophical disfavour—beautiful.^1^ The persistent interest in this question is certainly justified, given that beauty (as a quality that we ascribe to things or as a feeling that we experience) is a pervasive feature of our experience. We admire it in art as well as in nature, and we sense it in works of genius as well as with the most mundane objects—a face, a dress, the arrangement of furniture in a living room. Almost always, it seems, we are attributing or withholding this quality (or experiencing this feeling) with varying degrees of intensity. Now, one of the most persistent answers to the persistent question of beauty is that we find beautiful that which presents an *ordered arrangement of parts*. We find this idea, articulated in different forms, throughout the history of aesthetics, to the point that it is sometimes referred to as the ‘classic theory' or the ‘great theory' of beauty (see [3] for a useful summary). The idea, simply put, is that we experience beauty whenever we perceive that different elements in our sensorium (the notes of a musical piece, the pigments on a canvas, the features of a landscape) stand in ordered relationships with one another and conspire, as it were, to create a meaningful whole—a structure or pattern that we can grasp. Experiences of beauty, in other words, would be moments of sudden clarity about the structure of our world: moments where everything makes sense, everything clicks into place, everything is exactly as it should be, and more coherence, unity, consistency among disparate things is achieved. Artworks, in turn, would be objects specifically designed to afford these experiences. In this very simple characterization, this view encompasses features that have been linked to the experience of beauty and good art since antiquity, such as symmetry, harmony, balance and proportion [3,4]. It also encompasses formulae for beauty and artistic goodness of enduring influence in aesthetics, such as ‘organic unity' [5], ‘unity in variety' [6], ‘order in complexity' [7]. It is also behind much of the talk in aesthetics about form, formal unity, or formal perfection [8].^2^ Even more interestingly, the same idea is also common currency in contemporary psychological and neuroscientific research on the arts and aesthetics. Several psychologists and neuroscientists are in fact pointing out that the pleasurable experience that we associate with beauty or successful aesthetic encounters might be related to the discovery of patterns in our sensorium (see e.g. [10–17]). In fact, in one of the pioneering works in neuroaesthetics, Ramachandran & Hirstein [18] were already putting forward the hypothesis that (as Armstrong & Detweiler-Bedell [15, p. 311] aptly put it) ‘the brain rewards progress toward organizing the perceptual field into a meaningful configuration'. Artworks, then, according to them too, would afford this experience to an enhanced degree: they would highlight our ability to arrange our sensorium into meaningful configurations. These ideas in turn make contact with a Deweyan line of thinking, very much alive in contemporary philosophical aesthetics, according to which ‘aesthetics concerns everything that goes into our experience and creation of meaning, and the arts are recognised as exemplary cases of this pervasive process of meaning-making' [19, p. 225].

But the way in which we arrange our sensorium into meaningful configurations (the ‘pervasive process of meaning-making') is of course not the exclusive purview of artists and aestheticians. It is also, crucially, much of what psychology, neuroscience and cognitive science are interested in. In fact, it was already a central concern for the members of the Gestalt school in psychology. One of their key insights is indeed that, whenever we are presented with a sensory array, we tend to aggregate its various elements into wholes (Gestalts) that are as balanced, stable, and unified as possible [20]. In other words, if you see ‘your dog' and ‘your front door' in a sensory array, it is because these are the most stable and balanced wholes (Gestalten) under which you managed to group your current sensations. Some of the Gestaltists had even noted the relationship between this tendency towards ‘good' perceptual organizations and the pursuit of beauty. As Eysenck [21, p. 357] puts it (paraphrasing Koffka [22]): ‘perception is artistic. It will create the most symmetrical, the most balanced, the most beautiful mental picture which is possible under the external circumstances obtaining at the moment'. Koffka had also noted that deviations from ‘good' perceptual organization ‘hurt our sense of beauty' [20, p. 153]. Nowadays, however, the talk of Gestalt has been largely superseded (or rather productively engulfed in other paradigms: see [23]). But the attention towards the dynamic ways in which we give structure to our sensorium has of course never waned.

One increasingly popular way to capture this ability of ours is centred around the notions of inference, hypotheses and predictions. The idea is old [24–26], but has been given a new edge by recent neuroscientific approaches operating within the ‘Bayesian brain' hypothesis [27–30]. According to this idea, making sense of the stimulations impinging on our sensory organs means inferring or hypothesizing what structures in the world might have caused them. These structures might range from objects in the environment to causal structures linking events spanning longer spatial and temporal scales. In this picture, therefore, seeing our dog coming in through our front door means settling on the hypothesis that best explains the array of visual, auditory, olfactory stimuli currently impinging on our sensory organs; we do the same, on a higher level of abstraction, when we explain the mud on her pawns and the holes on our lawn as the product of a precise series of causally related events. These hypotheses are thought to become more or less probable as we interact with the environment and acquire new sensory evidence. According to a growing line of thinking, these oscillations in probability can be modelled with the tools of Bayesian probability [27–30]. This has led to a now widespread understanding of the brain as an organ of probabilistic inference, constantly trying to guess how the elements in its sensorium ‘hang together' at different levels of abstractions and spatio-temporal scales. These are, in fact, the conceptual foundations of predictive processing (PP), a theoretical framework in cognitive science that provides a neurobiologically plausible account of how this process of probabilistic hypothesis-testing might be carried out by the brain [31–34]. PP is the framework most papers in this theme issue refer to, and the one we now need to consider if we want to grasp all the implications of the encounter between aesthetics and cognitive science that this theme issue aims to establish.

PP (and neighbouring frameworks, such as ‘active inference’ and the 'free energy principle') was initially formulated as a general theory of brain function [31]. Like other Bayesian approaches, PP is grounded on the idea that the brain constantly tests probabilistic hypotheses, or predictions, against the incoming sensory stimulations. According to PP, these predictions unfold in a hierarchical fashion across many spatial and temporal scales, roughly following the hierarchical organization of cortical systems. A high-level prediction to see a dog, for example, may give rise to ‘lower-level predictions about limbs, eyes, ears and fur, which then cascade further down in terms of predictions about colours, textures and edges, and finally into anticipated variations of brightness across the visual field' [35, p. 108]. At each level of the hierarchy, predictions are compared with the sensory stimulations coming from the level below, and to the extent that there are mismatches between the two, ‘prediction error' signals are generated. These prediction errors are propagated up in the hierarchy and used to recruit new and better predictions, which are then compared again with the incoming sensory stimulations, and so on in an iterative fashion. This reciprocal exchange of bottom-up prediction errors and top-down predictions proceeds until at all levels prediction error is minimized and the brain has come up with the best available explanation of the data it is observing. In this way, by constantly reducing the mismatches between what it predicts and what it gets from the senses, the agent is able to preserve its viability as a model of its environment and make contact with a structured world full of objects, people and ordered events. PP provides, therefore, a rather elegant and articulate image of our meaning-making processes, one that, moreover, lends itself to computational modelling and empirical investigation. The real advantage of PP, however, is its ability to explain under the same conceptual apparatus many (some say all [32,33]) aspects of our mental functioning. In the last decade or so, the PP framework has in fact been expanded to account not just for perception and cognition but also action (which is seen as involving predictions about proprioceptive stimuli [36,37]) and emotion (which are seen as arising from predictions about interoceptive stimuli [38,39]). PP accounts have also been given for many other crucial psychological phenomena, including attention [40], affect and valence [41–43] curiosity [44,45], motivation [46], well-being [47] and a wide range of psychopathological conditions [48,49]. The result is a framework that aspires to account for all aspects of our mental life (and their disturbances) under a single explanatory principle: the brain's constant attempt to structure its sensorium at different spatial and temporal scales to maintain a grip on its world.

Having sketched out the specific understanding of both aesthetics and our mental functioning that interest us here, we are now in a position to see what is involved in the encounter between aesthetics and cognitive science that this theme issue showcases. On the one hand, we have a view, deeply rooted in the history of philosophy and very much alive in contemporary research, that connects beauty, art and aesthetics to the ways we make our sensorium coalesce into meaningful structures. On the other hand, we have a theory in cognitive science that describes the same processes and turns this description into a unified explanation of mental functioning. This leaves us with an understanding of aesthetics as a discipline that makes productive contact with—and in fact starts to look like—a general theory of meaning-making: a theory, that is, about how scattered stimulations coalesce into pleasing wholes, or, in PP terms, how we test probabilistic hypotheses about the structure of our environment. This line of thinking, we believe, opens up promising avenues of research for both aesthetics and cognitive science. Aesthetics can begin to frame its concerns about art, beauty and aesthetic experience within a broader interest for the dynamics of inference and meaning-making. In turn, cognitive science can start to profit from the wealth of insights about the dynamics of inference and meaning-making that philosophical aesthetics, the history of art and artistic practise have produced through the centuries, often disguised as mere aesthetic concerns. This is indeed the direction that the growing stream of research on PP and aesthetics is taking. While PP is increasingly adopted as a framework for understanding different artforms and aesthetic phenomena [50–56], the arts and aesthetics, examined under the PP lens, are starting to be seen as important windows into our mental functioning [45,52,57,58]. The result is a vast interdisciplinary research programme that promises to deliver important insights into our aesthetic encounters as well as a wide range of psychological phenomena of general interest, including perception, cognition, learning, affect, motivation, well-being and the dynamics of subpersonal and person-level experience. In the rest of this introduction, we point to some of the directions that this research programme is taking and the benefits that it could have for the different disciplines involved: philosophy and history of art (§2), psychology of aesthetics and neuroaesthetics (§3), and psychology and neuroscience more generally (§4).

## 2. Prospects for philosophical aesthetics and the history of art

We have said that the encounter between aesthetics and cognitive science that we are presenting aligns with age-old, deeply rooted philosophical intuitions about beauty, art and aesthetic experience. One might therefore wonder what new insights, if any, the philosopher and the humanist more generally stand to gain from this encounter. Granted: providing rigorous bases for connecting the study of the arts and aesthetics and the concerns of the cognitive scientist might already be a significant feat. It could lead, among other things, to a reconsideration of important moments or concepts in the history of aesthetics in light of present-day research in cognitive science. However, a doubt may still arise that what is at stake here is just a translation of old philosophical intuitions into a new neurocomputational language. One may also further worry that this translation aims to replace the rich picture of our aesthetic encounters that philosophers, artists and art historians have provided throughout history, instead of complementing or extending it in useful ways. This worry may in fact be reinforced by the rather colonial or reductionistic stance that some neuroscientific approaches to art and aesthetics have adopted in the past. In fact, a significant part of the early work in neuroaesthetics was carried out under the more or less explicit assumption that the subject matter of aesthetics will eventually yield to a reductionist description in terms of brain mechanisms.^3^ Such a line of thinking, often accompanied by surprisingly bold claims—such as that of having elaborated a ‘neurobiological definition of art' [59, p. 22] or having discovered ‘the key to understanding what art really is' [18, p. 17]—is responsible for the bad reputation that neuroaesthetics has among some humanists and for the strong criticisms that it has attracted [60,61].

We believe, however, that none of this is at stake in the encounter between PP and aesthetics. At the same time, it is certainly up to those who wish to articulate this encounter to deflect the above risks and avoid overly reductionistic drifts. Two things should be noted in this respect, and are starting to emerge clearly, we believe, from the growing stream of research in this area. The first is that, far from merely echoing well-established ideas, PP helps extend, sharpen and substantiate several interesting philosophical points about our aesthetic encounters. It also allows, we believe, to adjudicate between different philosophical views in the current philosophical and art-historical debate. The PP picture of our aesthetic encounters is, in other words, dense with theoretical implications (some of which, as we shall see, point directly to the limits of reductionistic approaches in aesthetics). PP itself is, after all, a theory currently at the centre of several philosophical debates, and one with a layered structure of claims and commitments that philosophers and humanists in general are scrutinizing and drawing inspiration from [62]. There is no principled reason to think that aesthetics should be left out of these discussions. The second thing to note is that, far from encouraging a reductionistic attitude that collapses historical/philosophical and neuroscientific levels of description, the PP picture has so far allowed philosophers, art historians and artists to enter into meaningful dialogue with psychologists and neuroscientists, a dialogue that includes informed disagreement (see e.g. [63,64]). Indeed, for this encounter between aesthetics and cognitive science to keep growing in complexity and explanatory potential, a certain productive friction between the two sides should be nourished and encouraged, so that both can bring the other into focus with benefits for the overall enterprise. Perhaps given the intuitive appeal of some of its basic apparatus, PP seems so far to have provided a useful framework for doing just that. Let us consider therefore some of the areas of debate where philosophers of art and aesthetics and art historians are profiting from PP and could profit more in the future.

A first philosophical issue where PP seems to offer new insights is the nature of aesthetic pleasure, or the question of why our aesthetic encounters are pleasurable, attractive and engrossing. The PP approach to this question leverages the link, crucial in PP, between perception and cognition (as processes that find structure in our sensorium) and fundamental existential concerns. Remember that in PP the agent is seen as an embodied model of the world constantly trying to preserve its viability in the face of an ever-changing environment (it is, as Hohwy puts it, a ‘self-evidencing' creature [65]). The oscillations in uncertainty in its hypotheses about the structure of its world are therefore direct signals about how well it is doing in ensuring its continued existence as a viable model of that world. This means that perception and cognition are always soaked with affect, tied as they are with the hope that the world will reveal some structure and further our existence [41–43]. What we call aesthetic pleasure, so the PP story suggests, is the positive affective feedback that we get when we are more successful than usual in making sense of our environment (or, in PP terms, in reducing prediction error; see [45] for a more detailed exposition). Aesthetic pleasure is, in other words, the mark of a cognitive and existential conquest. This makes for a complex picture, whose wider implications are still to be assessed, where aesthetic experiences are seen as both cognitive and affective, apparently gratuitous but at the same time tied up with deep existential needs. Such a picture might be brought into productive contact, we believe, with current discussions about the bodily and affective nature of our aesthetic encounters [66], as well as about classic notions in aesthetics such as that of ‘disinterested pleasure' [67].

Another related and very broad philosophical issue that PP seems to cast a new light on is the vexed question of the scope of ‘aesthetic experience' (and, consequently, the scope of aesthetics as a distinct philosophical endeavour). The history of aesthetics, as we have noted, is haunted by the question of what leads us to find certain things beautiful or aesthetically appealing; in more recent years, this has often turned into a question about what, if anything, sets ‘aesthetic experiences' apart from other, ordinary, mundane, ‘non-aesthetic' experiences (e.g. [68,69]). Positions in this regard range from claiming that the notion of aesthetic experience does not apply to anything, to restricting its scope to fairly narrow targets or situations (art or beautiful objects), to claiming (in a broadly Deweyan fashion) that ‘all meaningful experience is aesthetic experience' [19, p. 2]. This variety of diverging responses is evident also in the historical vicissitudes of aesthetics as a discipline, which has oscillated since its inception between being a philosophy of the arts, a theory of beauty and aesthetic experience, and a more general ‘*scientia cognitionis sensitivae*' (to use Baumgarten's [70] famous phrase). The PP take on the scope of aesthetic experience, however, is clear. A concern with beauty, aesthetic appreciation and aesthetic experience is also, by necessity, a concern with the dynamics underlying experience more generally. This is because, as we have seen, both aesthetic appreciation and the experience of a structured, meaningful world have to do with how scattered sensations coalesce into pleasing wholes. In this sense, therefore, ‘all meaningful experience is aesthetic experience', and experience is always, to some extent, aesthetic. At the same time, however, PP provides us with the tools to clarify why experience is not always aesthetic *to the same degree*, that is, why our everyday experience is not constantly imbued with the pleasure and awe that we tend to associate with paradigmatic aesthetic encounters. Remember that in the PP picture (as well as in many traditional approaches in philosophical aesthetics) aesthetic pleasure accompanies situations where we are *more successful than usual* in structuring our world. Most percepts fall short from providing this experience: they are either too orderly and predictable or too disorderly and unpredictable to allow us to discover patterns in an optimal way (i.e. to reduce prediction error to a significant degree). Only few percepts afford the right level of reducible ambiguity required to prompt a pleasurable moment of internal click. Even fewer provide a consistent, hierarchically organized flow of such moments capable of generating intensely pleasurable experiences that encourage prolonged engagement. Artworks, to the extent that they aim to be aesthetically effective, try to be this latter kind of percepts: they provide us with highly structurable sensory flows that ignite and sustain our meaning-making tendencies. In doing so, they promote experiences that, while made from the same building blocks of ordinary experiences (i.e. the same inferential processes by means of which we structure our sensorium), set themselves apart from the latter by the higher degree in which they mobilize and satisfy our inferential capacities. This line of thinking, we believe, makes justice to both the pervasive and the special character of our aesthetic encounters, acknowledging their continuity with ordinary experience while at the same time allowing for differences in intensity that are certainly there.^4^ It has also the added advantage of bringing more clarity and unity into aesthetics as a philosophical endeavour, reconnecting the study of art, beauty and ‘sensuous cognition’ under the same framework.

Within this broad, PP-inspired framework, many other philosophical questions about our aesthetic encounters can be productively explored. Many of these explorations will again profit from the clear link that the PP view establishes between aesthetic pleasure and deep existential concerns. In PP, as we have said, agents are seen as models of the world. A consequence of this basic assumption is that, in establishing the structure of the world at different levels of abstraction, they are also establishing the kind of creatures that they are. Each act of meaning-making is also, in other words, an act of self-discovery. If, as the PP view about aesthetics suggests, aesthetic encounters are moments where our meaning-making is particularly successful, then they are also moments of successful self-discovery: moments, that is, in which we determine both the structure of the world and of ourselves at a faster rate than usual. This might give us a way to understand, among other things, how artworks can fuel potent transformative experiences, what their cognitive value consists in [72], or why we may enjoy them even when they depict negatively-valenced content [73]. The same line of reasoning can also provide an argument against an overly reductionistic attitude in aesthetics: if artworks transform us in the way that the PP picture suggests, then a ‘neurobiological definition of art’ becomes a particularly illusory target to aim at; art, the PP picture makes clear, is effectively a means through which we escape definite determination and turn our minds and brains into moving targets (see [74] for a similar point). All these suggestions are of course speculative and will have to be fleshed out in detail before they can become informative. But they are, we believe, promising; they point once more to the philosophical import of a PP approach to aesthetics.

Apart from these broad philosophical indications about the nature of aesthetic experience, PP seems also well positioned to provide finer-grained insights into our engagement with the arts. These include suggestions about the kinds of predictions that inform the production and reception of particular artworks by particular audiences—a matter which art historians are chiefly interested in. In fact, seen from a PP perspective, the aims and interests of the cognitive scientist and those of the art historian tend to intersect. After all, both are interested in understanding what shapes our perceptual processes from the top-down, influencing our interpretation of the incoming sensory flow. What for the cognitive scientist are predictions or hypotheses that the agent makes about the structure of its world, for the art historian are conventions or expectations that inform our artistic and interpretive practices.^5^ Such expectations are likely to range from evolutionarily acquired expectations about the shape of our environment to culturally acquired expectations shared among the members of a community. In this sense, notions familiar to the art historians such as those of norm, genre, ‘schema' [75] or ‘script', can be interpreted as having to do with predictions acting at different level of the perceptual-cognitive hierarchy [76]. This, in turn, might lead to a reconsideration of classic art-historical debates about the role of the ‘beholder's share' [75] or the possibility of a ‘history of vision' [77] (see [51,57] for suggestions in this direction). Interesting possibilities in this area readily come to mind. One could, for example, examine from a PP perspective the ‘horizon of expectations' [78] of certain communities of observers to elucidate the reasons behind the good or bad reception of particular artworks or artistic styles (see e.g. [63] on the reception of contemporary conceptual art, and [79] on the reception of atonal music). In a similar vein, one could examine how different artistic styles or individual artworks shape and play with the different kinds of exteroceptive, interoceptive and proprioceptive predictions of their audiences (see [57] for some suggestions in this direction). Certain artworks (e.g. Impressionist or Cubist paintings) are likely to invite low-level hypotheses about what objects or subjects are depicted; other artworks (e.g. those with a clear iconographic character) might invite higher-level guesses about the role of the objects and subjects depicted in the overall symbolic economy of the work; still other artworks (e.g. Expressionist artworks) might invite guesses about what emotions the artist must have felt when generating them, or what movements she might have performed (think about Van Gogh's thick brushstrokes or Lucio Fontana's famous cuts on the canvas [80,81]); other artworks still (e.g. Dada, readymade art, Pop Art) are likely to play with our higher-level predictions about what artworks are supposed to look like according to the conventions of a particular ‘artworld'. The same conceptual apparatus (one that explains how we infer the hidden causes of our sensory data), could therefore, if used flexibly, illuminate the appeal (or failure) of very different artworks and artistic styles for different audiences.

## 3. Prospects for the psychology and neuroscience of aesthetics

We have seen how approaching art and aesthetics from a PP perspective can help the philosopher and the art historian shed light on important general questions about our aesthetic encounters, as well as narrower questions about art appreciation in specific contexts. However, at least since the second half of the nineteenth century, the study of the arts and aesthetics has ceased to be the exclusive preserve of philosophers and art historians and has gradually become an important object of study in psychology and neuroscience. ‘Empirical aesthetics' (this is the umbrella term that is often used to capture research in this area) is, in fact, one of the oldest strands of research in experimental psychology, having its roots in the pioneering work of Gustav Fechner [82]. Since its inception, it shared the core preoccupation of philosophical aesthetics with beauty and aesthetic appreciation, but approached it ‘from below' [82], starting from systematic empirical observations in an attempt to elaborate general theories. Over a century and a half, the discipline has evolved in multiple directions and around different theoretical frameworks (see [83,84] for useful overviews). The development of neuroimaging techniques in the 1990s added other key methodological tools to the field and led to the development of the subfield of neuroaesthetics, now an important area of inquiry in its own standing. Today, empirical aesthetics is a booming and varied area of research, with a growing presence in the scientific discourse and major recent collections of papers synthesizing its models and acquisitions [85–87]. The question is, then, what PP can offer to this vibrant area of research.

A good way of approaching this question is perhaps to look at the hopes and concerns voiced by researchers in the field. If one does so, one finds that the excitement for a field in rapid expansion is often accompanied by worries about the lack of agreement on its general underpinnings, its aims, and its results. In a 2021 opinion paper, Wassiliwizky & Menninghaus [88, p. 437] notice, for example, that ‘there is overall little agreement regarding the general conceptualization of empirical aesthetics as a distinct research field, the identification and definition of its key concepts, and a methodological framework for its future advancement'. In a similar vein, Carbon [89, p. 117] observes that ‘current research attempts are mostly unconnected to each other, even within one research group or even across different studies of one single researcher—they mostly lack ideas to connect different results and to comprise them by a more general theory on aesthetics'. The situation does not seem to be much different in the subfield of neuroaesthetics. Pioneered in the 1990s, it was described in 2014 as still in its ‘early days' [90] and in 2016 as ‘a relatively recent field of research' [91, p. 266]. In 2021, Chatterjee & Cardillo [86, pp. xii–xiii] still refer to it as a ‘very young field' and observe that researchers are ‘still establishing neuroaesthetics' conceptual underpinnings, the relevant scientific agenda, the optimal methods of inquiry and how best to engage with allied disciplines' (on the hesitancy of neuroaesthetics research in presenting its achievements, see also [92]). There is, in summary, a growing awareness of the need for empirical aesthetics to define its conceptual framework, its specific aims, its methods and its relationship with other disciplines; there is also a need to bring research done within different frameworks and approaches ‘into a unified paradigm' [88, p. 437], a paradigm that, ideally, can also explain scattered (if not seemingly inconsistent) findings in the field. Our impression is that current research on PP and aesthetics offers suggestions (no doubt tentative and to be further articulated) about how to meet most of these pressing needs.

Let us say a few words on the first issue: how PP can help empirical aesthetics clarify its conceptual underpinnings. PP, as we have said, is rooted in Bayesian cognitive science. It leverages the increasingly popular understanding of the brain as an organ of probabilistic inference and generalizes it to explain many different aspects of mental functioning. As a conceptual framework for the study of aesthetics, it has several appealing features. First, as we have seen, it provides a neat account (corroborated by age-old philosophical intuitions) that connects aesthetic pleasure with more pervasive processes of inference and meaning-making and their underlying existential concerns. This account allows us to specify what kind of percepts engender more aesthetic pleasure (namely, those that allow for more reduction in prediction error) and why (because they ensure our viability as models of the world). Second, PP allows us to apply this general account  in a fine-grained way to particular stimuli and specific artforms (thanks to the intuitive appeal of notions like ‘predictions' and ‘hypotheses', which, as we saw, have antecedents in the philosophy and history of art). Third, PP also allows us to model the dynamics of our engagement (thanks to its Bayesian apparatus) and to connect these dynamics with known facts about brain function (thanks to its hypotheses about neural implementation). More of course will have to be said about how exactly ‘predictions' are involved in specific cases, but the work done so far seems at least to suggest that the PP apparatus is plastic enough to be applied productively to a wide variety of artforms, including visual art [50,51,57,93], music [52,54], literature [53,94,95], cinema, [55,56,96] and games [97].

What about the aims of empirical aesthetics as seen from a PP perspective? Here, as we saw, the PP picture also introduces clarity. The concern with beauty, aesthetic appreciation and aesthetic experience is to be framed within a general concern with the dynamics of inference and meaning-making. This means that the attempt to clarify aesthetic phenomena can benefit from the study of these broader dynamics and does not necessarily have to rely on the examination of the range of stimuli normally labelled as ‘aesthetic' (see [98] for other suggestions in this direction). It also means that the study of aesthetics can in turn provide insights into the dynamics of inference and meaning-making in general (more on this in §4). This position, we believe, has the advantage of maintaining the specificity of empirical aesthetics as a distinctive field of inquiry without at the same time isolating it from the rest of psychology and neuroscience, but rather making it an important part of those broader endeavours.

What about the best methods of inquiry? Here, the PP approach to empirical aesthetics is more liberal, but (perhaps for this very reason) also more imprecise. PP is, as we noted, a theory with a layered structure of commitments. As a general theory of mental functioning aiming to unify a wide spectrum of psychological phenomena, PP lends itself to be tested with many of the methods and paradigms of mainstream experimental psychology. But as a theory about brain function that makes tentative mappings between inferential processes and brain dynamics, PP also lends itself to be tested with many of the methods of mainstream cognitive neuroscience. At both levels, however, a major challenge for the PP approach (inside and outside aesthetics [99]) is to make its conceptual apparatus sharp enough to allow differentiation between its predictions and those of alternative models. In aesthetics, this would mean trying to differentiate PP hypotheses from those views pointing to the role of ‘predictions’ or ‘expectations' in aesthetic appreciation in a rather broad and untechnical sense. The challenge is, in other words, to isolate with precision the predictions and prediction errors (i.e., the ebbs and flows of uncertainty about the structure of our sensorium) in the individual, experiencing brain. This challenge is all the more formidable in the case of our engagement with artworks, since the predictions that individuals bring to bear on such complex stimuli are often highly idiosyncratic, deeply entangled with one another, and (most of the times) not directly observable in terms of brain dynamics. As a result, as long as we do not have reliable methods to identify prediction and prediction error activity in the brain with sufficient spatial and temporal resolution, we are hampered in testing an account—like the PP account of our aesthetic encounters—that links these neural dynamics to features of our experiences such as curiosity, insight, and pleasure (but see e.g. [14,54] for reviews of the attempts in this direction). While we await significant advances in tracking these dynamics in the brain, however, there are several viable workarounds that allow to test various aspects of the PP proposal. Many of them rely on well-crafted behavioural paradigms, preferably coupled with manipulations of uncertainty or explicit computational (Bayesian) modeling. A solution that can be used to some effect, for example, is to use crowdsourced measures of uncertainty (computed based on the entropy of the distribution of responses of a large group of participants) to predict the strength of the relevant responses, such as curiosity, insight, or pleasure (see e.g. [49,100]). Another interesting solution is the one adopted by Cheung and colleagues [101,102]), who used uncertainty and surprise measures derived from a machine-learning model trained on a large corpus of popular songs to predict the pleasure experienced by participants while listening to chord progressions (an approach that could be extended, in principle, to pose finer-grained questions about the enjoyment of particular genres by audiences with different degrees of expertise). Beyond music, large language models trained on particular styles of texts may provide similar proxy quantifications of uncertainty and surprise to predict text-induced curiosity, insight and pleasure (see e.g. [103] for useful indications). These and other inventive behavioural, computational, and neurocognitive paradigms will hopefully allow us to go beyond blanket claims that ‘predictions are important in aesthetic experience' and help us track with ever greater granularity the complex meaning-making dynamics at play during our aesthetic encounters.^6^

On the other hand, a certain openness to the not-immediately-testable seems required to effectively connect the level of brain dynamics and empirically testable phenomena with the phenomenological, social and historical levels of analysis that interest philosophers, artists and art historians. In other words, if the PP picture of our aesthetic encounters is to be of any use, it should make room for the idea that not all the ‘predictions' or ‘hypotheses' involved in our active engagement with an object of aesthetic appreciation might be capturable in specific constructs or studied with the empirical paradigms currently at our disposal. This should not, however, restrict the theory-building of the empirical aesthetician, but rather open her to that ample reservoir of non-quantitative evidence about the dynamics of our experience that the artist, the aesthetician, and the art historian can provide (more on this, again, in §4). Only in this way, we believe, will empirical aesthetics integrate not only within psychology and neuroscience, but also with sister humanistic disciplines with a keen interest in the same topics.

We have seen therefore that PP might help empirical aesthetics address broad questions about the field's conceptual underpinnings, aims, methods and dialogue with other disciplines. We have not said anything yet about how the PP approach to aesthetics scores when it comes to explaining the large amount of evidence that empirical aesthetics has produced since its inception. This is a crucial point, of course: if the PP story about aesthetics cannot accommodate many classic findings in the field, its significance would be diminished. A systematic attempt to bring the PP picture in contact with the major frameworks and results in empirical aesthetics is still missing, but what emerges from the existing discussion on the topic (e.g. [7,45,106–109]) is encouraging. It seems to indicate that PP can indeed enter into productive contact with—and in fact incorporate—a good part of past and present research in the field. The PP view, as we have seen, suggests that aesthetic pleasure accompanies moments when we are particularly successful in finding structure in our sensorium. This same idea, we said, was present in philosophical aesthetics from the beginning. But the same is also true of empirical aesthetics. Fechner's ‘principle of the unified connection of the manifold' [82] can be readily read in these terms. This is also not dissimilar, as we saw, from what the Gestaltists were noticing when they pointed out that perception is ‘artistic’, as it tends to form the best (most orderly, balanced and unified) patterns available given the circumstances [21,22,110]. By the same token, PP can also accommodate the apparent contrast between two broad families of theories that have opposed themselves throughout the history of empirical aesthetics: namely, those positing a preference for percepts with an intermediate level of complexity (or entropy, unpredictability, etc.) [111], and those positing a preference for stimuli that are as simple, prototypical, repeated, fluently processed as possible [112–114]. From a PP perspective, the contrast between these two families of theories is only apparent and stems from an undue emphasis given, respectively, to the starting point and the endpoint of what should really be conceived as a process of successful structuring: if we seem to prefer stimuli that are not completely structured, it is because these allow for more structuring further down the line, and we are confusing the starting point with the process; whereas if we appear to prefer stimuli that are already well structured, it is because we are focusing on the endpoint of the structuring process that led us there (see discussion in [45] on this point). For its stress on the process of successful structuring, PP is also in line, as we saw, with many recent theories that point to a role of pattern-finding, or learning, in generating aesthetic pleasure [10–16]. Finally, PP seems also compatible with approaches pointing to a role of embodied motor simulation in generating aesthetic pleasure [80,81]: from a PP perspective, actions are inferred like any other distal cause of sensory stimulations, so inferring them can be, like any other inferential process, pleasurable. Of course, these brief and sparse remarks are not meant to prove anything. Seeing whether the PP account really explains any of the past or present acquisitions of empirical aesthetics will require a careful examination aimed at verifying whether the predictions of the PP story are really in line with the existing empirical evidence, as well as devising new experiments that test specific PP hypotheses. It will also require attention to other factors that enter the aesthetic exchange, such as context [115], expertise [17], and personality traits [116] (all, we believe, in principle tractable from a PP perspective). Even at this stage, however, it seems that PP has at least some potential to unify different strands of previous literature in the field and ensure a further move towards a more unified empirical aesthetics.

## 4. Prospects for psychology and neuroscience in general

In the previous two sections, we have focused on how the PP framework could benefit and propel forward the interdisciplinary study of aesthetics. However, right from the outset we made clear that what is at stake in this encounter between aesthetics and cognitive science goes beyond the mere clarification of the first by means of the latter. In fact, the picture that we have painted suggests that the two should really be seen as partners in the same endeavour: that of exploring the complex, dynamic and precarious ways in which we make the world coalesce into meaningful structures, with all the affective and existential implications that this entails. In this sense, the ambition of those approaching art and aesthetics from a PP perspective should not so much be to elaborate a science of art and aesthetics, but rather to set a framework in which art and aesthetics can collaborate with psychology and neuroscience in the study of crucial features of the human mind. Such an enterprise would certainly not be new (see e.g. [117–119]), but, thanks to PP's wide-ranging explanatory ambitions, it could acquire a new significance and breadth of aims. If PP can really provide a viable unified account of our mental functioning (including perception, cognition, action, emotion, affect, learning and attention among other phenomena), and if there is a clear link between the PP account and the concerns of the arts and aesthetics, then the study of the arts and aesthetics can also deliver insights about all the above phenomena. The arts and aesthetics, in other words, would gain a principled way to illuminate many of the crucial topics of interest for psychologists and neuroscientists. Efforts in this direction are already underway: three lines of research in particular, we think, are starting to be pursued and to yield promising results.

The first line of research has to do with the exploration of the dynamics of (subpersonal and person-level) experience. Remember that in the PP picture making sense of the world means testing probabilistic hypotheses against incoming sensory stimulations at multiple temporal scales. These hypotheses lose and gain in probability as we acquire new sensory evidence, giving our meaning-making its characteristic uneven course. Many of these dynamics unfold too quickly and automatically to be consciously experienced, but others have clear phenomenal correlates (think of the sense of closure and discovery that you get when you finally see a pattern in an ambiguous visual array). The crucial step is to recognize that artists (and also aestheticians, art historians and art critics to some extent) possess a sophisticated albeit largely implicit understanding of these dynamics. They can feel, for example, the visual tension of an unbalanced picture; they can tell what note or word is the right one to close a melody or a sentence; they can orchestrate the ups and downs of tension of a suspenseful narrative; they can produce a cinematic flow where the editing is predictable or unpredictable, invisible or marked. More generally, they know what cues ought to be presented to our sensory organs for us to formulate certain evolving hypotheses about what we are sensing.^7^ In other words and in PP terms, artists possess a complex understanding of the dynamics of inference. What is more, throughout history, they (and aestheticians, art historians, etc.) have also captured and formalized such an understanding in treatises and manuals (about painting, music theory, rhetoric, storytelling, etc.), which under the proper lenses can be seen as important sources on the processes through which we structure our sensorium. Some of this knowledge is even explicitly expressed in probabilistic terms.^8^ What PP adds to this body of knowledge is a Bayesian apparatus that models the same dynamics that artists intuitively master and aestheticians try to describe and link them in plausible ways with facts about brain function. This happy encounter between the rich phenomenological intuitions provided by artists and aestheticians, Bayesian computational modelling, and the study of neural dynamics is in fact starting to deliver insights particularly in music research (see e.g. [52,54,101]), which already possesses both a rich corpus of intuitions about phenomenal effects (i.e. music theory, which has been described as ‘the most formally developed example of a folk psychology currently extant' [121, p. 645]), effective tools to model inferential dynamics [122], and a characteristic set of electrophysiological responses [52]. But the same approach could in principle be extended to all the arts (see e.g. [93,123] for some preliminary suggestions about visual art, [53,94] about literature and [55,56,96] about cinema) and even beyond exteroceptive inference to embrace proprioceptive and interoceptive inference as well. By revealing systematic connections between aspects of our phenomenology and facts about neural dynamics, this line of research might well contribute to research in ‘neurophenomenology' [124] and even inform theoretical discussions about consciousness from a PP perspective (see [64,125] for preliminary suggestions in this direction).

The second, related line of research takes full advantage of the link that PP establishes between inference and affect. In the PP picture, as we have said, our guesses about the structure of the world are not probabilistic calculations carried out in a cold, indifferent manner. Rather, they are always imbued with affect and related with deep existential concerns: what gains or loses terrain together with these guesses in a dynamic fashion is, after all, our own existence as viable models of the world. Our experience is therefore always tinged with a myriad of subtle and varying positive and negative affective nuances related to how well we are doing in our attempts to make sense of the world (what are sometimes called ‘epistemic emotions' or ‘metacognitive feelings' [126,127]). These include feelings like surprise, confusion, curiosity, uncertainty, boredom, insight and (dis)fluency, many of which have been discussed from a PP perspective [42,44,45,128,129]). Now, if the PP view about art and aesthetics is on track, the artists' implicit understanding of the dynamics of inference is also, necessarily, an understanding of the dynamics of affect. In other words, artists are not just astute manipulators of our hypothesis-testing processes, but also of the affective reverberations that these processes have on us as embodied models of the world. In fact, in the PP picture, aesthetic pleasure itself is an affective correlate of successful inference: it is, as we saw, the feeling of having attained a particularly good explanation of the causes of sensory stimulations. The fact that skillful artists can engender this pleasure, therefore, betrays an implicit understanding of the dynamics necessary to produce it. Of course, however, artists know more than just how to engender this pleasure (and that is what makes the PP story about aesthetics something more than just a narrow theory about pleasure or liking): they also know how to cause puzzlement, surprise and disconcert, how to generate and sustain our curiosity and our motivation to keep engaging on the look for meaning, how to finally make us grasp some meaning and close our epistemic arcs, and how to organize these arcs in complex series and embed them in intricate hierarchical structures [130]. These sophisticated abilities constitute another indication of the artists' expertise in affect and affective engineering. Psychologists and neuroscientists moving within the PP approach are starting to use this expertise for their theoretical and empirical work (see e.g. [55,56,97,131–133]). Here again the insights of artists and aestheticians, complemented by the Bayesian apparatus of PP, promise to deliver fine-grained pictures of how feelings like confusion, surprise, curiosity, boredom, insight, etc., are generated, evolve over time, and can be managed by the sort of designer environments we humans build and inhabit. The acquisitions from this line of work could then add an interesting affective twist to broader debates about cognitive extension and niche construction [134]. They could also inform current discussions about ‘affective scaffolding' [135] and ‘extended affectivity' [136], some of which already see art as an important case in point [137,138].

The third line of research, which stems quite naturally from the previous two, consists in exploring the consequences of this encounter between PP and aesthetics for our understanding of optimal and suboptimal psychological functioning. In fact, in the PP story, aesthetic pleasure is tied in a fundamental way with optimal functioning: it is the feeling that we get when we manage to effectively structure our sensorium and preserve our viability as embodied models of the world. It is also the feeling that orients us towards portions of the world that can be effectively structured and away from portions of the world that cannot (because they are already clearly structured or unstructurable), furthering our growth and development. In this respect, the PP story about aesthetics is just confirming certain long-standing hypotheses about the kind of experiences that facilitate learning and human flourishing [11,139–141]. If this story is on track, then, the arts and aesthetics have something to say not just about the dynamics of inference and affect, but also about how these dynamics can be harnessed to design experiences that favour involvement, motivation, fulfilment, learning and discovery. They can teach us how to practise and encourage virtuous forms of epistemic behaviour, ones that keep us open and receptive towards environmental uncertainty and avoid that we get stuck in our own constructions or close our hypothesis-testing too soon or too permanently (see [98,142] for preliminary suggestions on how art and aesthetics might accomplish these feats). At the same time, and by contrast, art and aesthetics could have something to say about those cases in which our self-evidencing goes awry. These include ‘pathologies’ of our individual and collective epistemic behaviour of particular social relevance such as confirmation bias, echo chambers and conspiracy theories, all of which are starting to be examined from a PP perspective [143,144]. However, they also include many psychopathologies such as schizophrenia, delusions, autism spectrum disorder, anxiety disorders, depression, post-traumatic stress disorder, obsessive–compulsive disorder and addiction, all of which are also increasingly conceptualized within a PP perspective as anomalies of some kind in exteroceptive, interoceptive or proprioceptive inference (see [47–49] for useful summaries and discussions). In all these cases, PP suggests, something goes wrong with our attempts to manage environmental uncertainty and remain viable models of the world (even if in some cases these supposed pathological or maladaptive behaviours are perhaps better seen as proactive attempts to recover that viability). By providing images of what successful coping with the environment looks like, art and aesthetics could then illuminate the various pathological breaches in this process, clarifying what they consist in, why we indulge in them, and how some of them could be attenuated. In turn, bringing both art and psychopathology under the same PP lenses could point to new avenues of research on the therapeutic effects of art and creative activities [145,146], as well as clarify the long-debated relationship between creativity and mental illness [147].

## 5. Concluding remarks

We have arrived, we believe, at a picture with several appealing features. We did so by starting from a view of our aesthetic encounters firmly rooted in the history of philosophy and actively pursued in contemporary research. We then noticed how this view makes contact with an ambitious general explanation of our mental functioning. This contact, we suggested, offers to the philosopher and the art historian new possibilities of theoretical exploration, and provides the field of empirical aesthetics with new underpinnings and a way to synthesise many of its past and present acquisitions. More generally, we suggested, this contact offers a principled way for aesthetics and cognitive science to inform one another and contribute to the clarification of important psychological phenomena.

The reader will hopefully find developments of all these intersecting lines of research in the other contributions of this theme issue. The first part of the issue, entitled “General Issues”, gathers contributions that clarify the conceptual bases of the encounter between PP and aesthetics and expand it in new directions [98,142,148,149]. The three parts that follow—devoted to “Visual Art” [63,123], “Music” [102,132,133] and “Literature, Narrative and Cinema” [56,95,96,125] respectively—offer an articulate picture of the insights that PP can provide when applied to different art forms (including some that have so far been little or never explored from a PP perspective), and what in turn these art forms, when considered from a PP perspective, can tell us about our mental functioning. The last part, entitled “Responses and Critical Perspectives” contains papers that compare the PP picture of our aesthetic encounters with other leading proposals in the field and provide useful criticisms and indications for future research [64,107,108].

Taken together, the contributions of this theme issue go some way, we hope, in articulating the wide-ranging but hopefully coherent research programme that we have described — a programme that, as we noted several times, is certainly still in its infancy, with most of the relevant theoretical and empirical work lying ahead. While the empirical fate of PP as a general theory of mental functioning and as a framework for understanding our aesthetic encounters will surely become clearer in coming years, what is important now is whether the research programme that we have outlined is “progressive” or “degenerating”, to use Imre Lakatos' famous distinction [150]. In degenerating programmes, as Lakatos described them, “theories are fabricated only in order to accommodate known facts” [150, p. 5]. Arguably this is, at least for now, not the case for PP. On the contrary, compared to several other explanatory endeavours in aesthetics, PP tries to reason from a very limited set of principles for general cognitive and organismal functioning, without creating additional ones to account for our aesthetic encounters or any of the varied empirical findings in this field. A progressive research programme, on the other hand, leads us to new and unforeseen predictions. It does not just offer a redescription of known facts in its own terms, but rather opens up new hypotheses that inform new observations, and so on in a virtuous cycle. We have tried to suggest that the programme generated by the encounter between PP and aesthetics is of this latter kind. Our hope is therefore that the reader leaves this introduction (and the theme issue as a whole) not convinced of anything in particular, but with a sense of possibilities to be explored and structures to be found.

## Data Availability

This article has no additional data.

## Endnotes

1 According to some, aesthetics should stop worrying about beauty altogether (because the notion is too vague, limited or compromised) and concentrate instead on the study of ‘aesthetic experience’ [1,2]. Sometimes this move seems more verbal than substantial, and aesthetic experiences seem to reduce to what others would have called experiences of beauty. Some other times the move has more substantive reasons. From our perspective, in any case, the two enterprises tend to overlap, for reasons that we will outline.

2 On the notion of formal perfection see e.g. Cochrane [9, p. 31], who notices: ‘It is widely acknowledged that beautiful things… display formal perfection… something has form when it has discernible parts that bear some kind of relation to each other (be it conceptual, material or functional). The object then shows formal perfection when these parts relate to each other in a definitely ordered way; a way that makes sense’.

3 Semir Zeki, for example, maintains that ‘aesthetic theories will only become intelligible and profound once based on the workings of the brain’ [59, p. 217].

4 The position has also the advantage of providing an answer to a persistent worry that philosophers have about neuroscientific approaches to the arts and aesthetics (see e.g. [60,61]). In the words of Carroll *et al*. [71, p. 51]): ‘If the nature of art can be explained in terms of ordinary psychological processes subserving our engagement with artworks, one needs an additional explanation to determine what, if anything, differentiates artworks from ordinary artifacts’. In the PP picture, the basic processes are the same (i.e. processes of probabilistic inference), but there is still a difference in how these are leveraged and mobilized in paradigmatic aesthetic encounters, such as our engagement with effective artworks.

5 This focus on top-down inferences might be another reason to think that PP avoids some of the pitfalls of previous neuroscientific approaches to art and aesthetics, which have often focused on bottom-up, stimulus-driven and context-insensitive processing (see [57] on this point).

6 Other problems in testing the PP proposal can also be addressed by careful experimental design. For example, the problem of not knowing which predictions participants will apply can be circumvented by inducing new predictions in the lab (i.e., by creating new regularities in the experimental setting) instead of working with pre-existing ones. The strength of the induced predictions can then be manipulated systematically, by manipulating the reliability of the regularities in question. In subsequent phases, relevant affective experiences (curiosity, insight, pleasure, etc.) can be measured for violations of (high- vs low-precision) predictions, and for violations that allow subsequent recovery of predictability. The PP account would predict a more negative experience for the first condition (compared with a “business-as-usual” predictable sequence as control condition), but a more positive experience for the latter (compared to the same control condition). While no study has explicitly followed this formula so far, there is independent evidence that inconsistencies evoke negative affect [104] and resolutions of uncertainty evoke positive affect [105]. Relatively simple behavioural paradigms like these will likely continue to reveal determinants of affective responses that very relevant to our understanding of our aesthetic encounters.

7 Seth [57, p. 385] puts this quite nicely when he observes, about a painting by Pissarro: ‘Pissarro's talent lies in ‘reverse engineering’ the visual system, to recover the afferent sensory signals that trigger a particular cascade of perceptual inference, rather than depicting the outcome of this process. Impressionist paintings can therefore be understood as a series of experiments into the inferential operations of the visual system and – more broadly – into the nature of the subjective experiences entailed by these operations. These artistic “experiments” complement contemporary neuroscientific attempts to reveal how top-down perceptual predictions underpin visual experience’.

8 Music is a good case in point. According to Meyer [120, p. 414], for example: ‘That musical styles are internalized probability systems is demonstrated by the rules of musical grammar and syntax found in textbooks on harmony, counterpoint, and theory in general. The rules given in such books are almost invariably stated in terms of probability. For example, we are told that in the tonal harmonic system of Western music the tonal chord is most often followed by the dominant, frequently by the subdominant, sometimes by the submediant and so forth’.
